# Impact of Dexamethasone on Three-Dimensional Stem Cell Spheroids: Morphology, Viability, Osteogenic Differentiation

**DOI:** 10.3390/medicina61050871

**Published:** 2025-05-09

**Authors:** Heera Lee, Ju-Hwan Kim, Hyun-Jin Lee, Jun-Beom Park

**Affiliations:** 1 Department of Periodontics, College of Medicine, The Catholic University of Korea, Seoul 06591, Republic of Korea; yysmjj@naver.com (H.L.); juhwank33@naver.com (J.-H.K.); hyunjinlee0423@gmail.com (H.-J.L.); 2 Department of Medicine, Graduate School, The Catholic University of Korea, Seoul 06591, Republic of Korea; 3 Dental Implantology, Graduate School of Clinical Dental Science, The Catholic University of Korea, Seoul 06591, Republic of Korea

**Keywords:** cell survival, cell differentiation, cellular spheroids, dexamethasone, osteogenesis, stem cells

## Abstract

*Background and Objectives*: Dexamethasone has been widely researched for its ability to promote osteogenic differentiation in mesenchymal stem cells in basic research. This study focused on examining the effects of dexamethasone on both cell viability and osteogenic differentiation in three-dimensional stem cell spheroids. *Materials and Methods*: These spheroids were created using concave microwells and exposed to dexamethasone at concentrations ranging from 0 μM to 100 μM, including intermediate levels of 0.1 μM, 1 μM, and 10 μM. Microscopic analysis was used to qualitatively assess cellular viability, while a water-soluble tetrazolium salt-based assay provided quantitative viability data. Osteogenic differentiation was evaluated by measuring alkaline phosphatase activity and calcium deposition using Alizarin Red staining. Additionally, the expression levels of genes associated with osteogenesis were measured through quantitative polymerase chain reaction. *Results*: The spheroids successfully self-assembled within the first 24 h and maintained their structural integrity over a seven-day period. Analysis of cell viability showed no statistically significant differences across the various dexamethasone concentrations tested. Although there was an observed increase in alkaline phosphatase activity and calcium deposition following dexamethasone treatment, these differences were not statistically significant. RUNX2 gene expression was upregulated in the 1 μM, 10 μM, and 100 μM groups, while COL1A1 expression significantly increased at 0.1 μM and 1 μM. *Conclusions*: These results indicate that dexamethasone supports cell viability and enhances RUNX2 and COL1A1 expression in stem cell spheroids.

## 1. Introduction

Dexamethasone, a synthetic glucocorticoid, is commonly used in stem cell research for its potent anti-inflammatory and immunosuppressant properties [1,2]. Dexamethasone can influence various aspects of the behavior and the differentiation of stem cells [3]. In tissue engineering, dexamethasone is often used to enhance the formation of bone and cartilage tissues [4]. Dexamethasone promotes the synthesis of extracellular matrix components, including collagen type I and bone sialoprotein, during the process of osteogenesis [5]. Dexamethasone influences the expression of genes associated with osteogenic differentiation, including RUNX2 and SOX9 [4,6].

Stem cells have recently garnered significant attention, particularly in research and the medical field [7,8,9]. They are a type of undifferentiated cell capable of both self-renewal and differentiation into specialized cell types [7]. Owing to these abilities, stem cells have attracted considerable interest for their potential to repair injured tissues and treat a range of diseases [10]. They are commonly classified based on their capacity for self-renewal, differentiation, and their source. Adult stem cells, such as mesenchymal stem cells (MSCs), exhibit multipotency, allowing them to differentiate into various cell types of their tissue of origin [11]. Conversely, induced pluripotent stem cells are adult cells that have been genetically reprogrammed to exhibit pluripotency, enabling differentiation into any cell type in the body [12]. Stem cells possess the capability to replenish or restore damaged tissues and organs [13]. Additionally, they can be applied to develop models for drug testing or disease modeling [9,14,15]. A three-dimensional stem cell-based model enables an efficient and reproducible formation of spheroids, which maintain structural integrity over several weeks and demonstrate enhanced osteogenic differentiation compared to conventional two-dimensional cultures [16]. The combination of stem cells with molecules such as growth factors has been reported to enhance bone regeneration and angiogenesis [17,18]. To undergo vigorous osteogenic development in vitro, mesenchymal stem cells require additional agonists [19,20]. The use of dexamethasone on MSCs has also been shown to regulate immune responses by influencing interactions between MSCs and immune cells [21]. Specifically, dexamethasone-treated MSCs can suppress immune cell proliferation and modulate the behavior of T cells and macrophages [22,23].

Dexamethasone, a synthetic glucocorticoid, is widely recognized for promoting osteogenic differentiation by regulating key transcription factors such as RUNX2 and enhancing the production of extracellular matrix proteins, including type I collagen. In conventional two-dimensional (2D) cultures, dexamethasone has been shown to induce osteoblast differentiation and matrix mineralization. However, its effects within three-dimensional (3D) spheroid systems, particularly those formed from gingiva-derived mesenchymal stem cells (GMSCs), remain insufficiently characterized.

Given that 3D spheroid models more closely replicate the native cellular microenvironment and promote enhanced cell–cell and cell–matrix interactions, evaluating the impact of dexamethasone in this context is essential for optimizing stem cell-based regenerative therapies. Therefore, the present study aimed to investigate the effects of dexamethasone on the cellular morphology, viability, osteogenic potential, and gene expression of GMSC spheroids, providing novel insights into its potential applications in bone tissue engineering and regenerative medicine.

## 2. Materials and Methods

### 2.1. Generation of Cell Spheroids Using Gingiva-Derived Mesenchymal Stem Cells

The Institutional Review Board at Seoul St. Mary’s Hospital, College of Medicine, The Catholic University of Korea, granted approval for the research protocol (KC22SISI0513) on 22 August 2022. Mesenchymal stem cells were extracted from gingival tissue and processed using established methods for isolation and characterization [24]. Following initial seeding on culture dishes, cells that did not adhere were removed, and the culture medium was refreshed every two to three days. For the creation of spheroids, commercially available concave microwells (H389600, StemFIT 3D; MicroFIT, Seongnam-si, Gyeonggi-do, Republic of Korea) were used. Each microwell was populated with one million stem cells, facilitating spheroid formation through cell-to-cell adhesion. To assess its effect on spheroid development, dexamethasone was applied in concentrations ranging from 0 μM to 100 μM, including intermediate levels of 0.1 μM, 1 μM, and 10 μM. The morphological characteristics of the spheroids were observed and evaluated on Days 1, 3, 5, and 7.

### 2.2. The Assessment of Cellular Viability

Stem cell spheroids were cultured in osteogenic media, with evaluations conducted at predetermined intervals. Qualitative assessments of the spheroids were carried out on Days 1 and 3 using a dual-color assay designed to detect plasma membrane integrity and esterase activity, utilizing propidium iodide and calcein acetoxymethyl ester (Live/Dead Kit assay, Molecular Probes, Eugene, OR, USA) [25]. Furthermore, quantitative analysis of cellular viability was performed on Days 1, 3, 5, and 7 using a water-soluble tetrazolium salt-based assay (Cell Counting Kit-8, Dojindo, Tokyo, Japan) [26]. For each condition and time point, three technical replicates were evaluated.

### 2.3. Quantification of Alkaline Phosphatase Activity and Calcium Deposition

Osteogenic differentiation was evaluated by measuring alkaline phosphatase (ALP) activity and quantifying calcium deposition using an anthraquinone dye-based method [27]. Stem cell spheroids cultured under osteogenic conditions were collected on Days 7 and 14. ALP activity was assessed using a commercially available assay kit (K412-500, BioVision, Inc., Milpitas, CA, USA). Calcium deposition analysis was conducted on Days 7 and 14 employing an anthraquinone dye assay [28]. Prior to staining, the spheroids underwent sequential washing and fixation. Alizarin Red S staining was subsequently applied at room temperature for 30 min. Following the staining procedure, the bound dye was extracted, and its concentration was determined using cetylpyridinium chloride.

### 2.4. Total RNA Extraction and Real-Time Polymerase Chain Reaction Quantification

RNA was extracted to assess its yield and integrity. The concentration of RNA was determined through spectrophotometric analysis (ND-2000, Thermo Fisher Scientific, Inc., Waltham, MA, USA) by measuring absorbance at 260 nm and 280 nm. To quantify mRNA expression levels, real-time quantitative polymerase chain reaction (qPCR) was conducted [29]. The primer sequences employed in this study are provided in Table 1. β-actin was utilized as an internal reference for normalization.

### 2.5. Statistical Analysis

Statistical data were presented as mean values with corresponding standard deviations for each experimental group. To ensure the validity of statistical assumptions, assessments of normality and homogeneity of variance were conducted to verify that the dataset conformed to a normal distribution and exhibited consistent variance. The experimental results, influenced by dexamethasone concentration and time points, were analyzed using a two-way ANOVA. Furthermore, groupwise comparisons were performed using a one-way ANOVA, followed by Tukey’s post hoc test, with all calculations executed using SPSS 12 (SPSS Inc., Chicago, IL, USA). A significance level was established at *p* < 0.05.

## 3. Results

### 3.1. Generation of Spheroid-Shaped Stem Cell Aggregates

**Figure 1:**
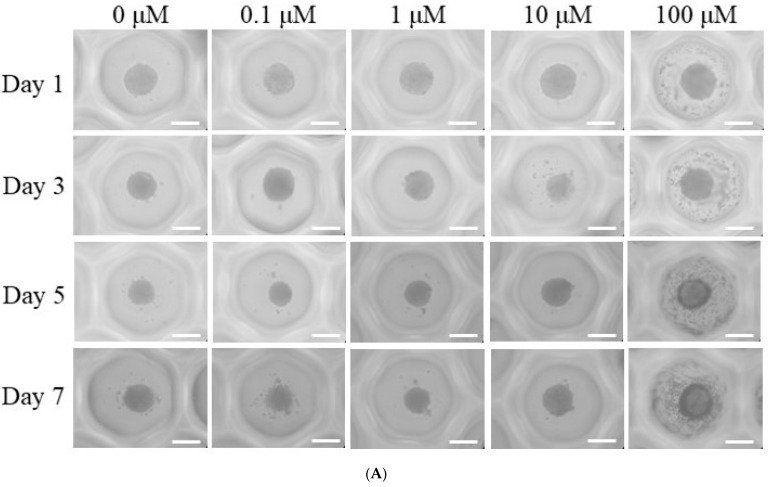

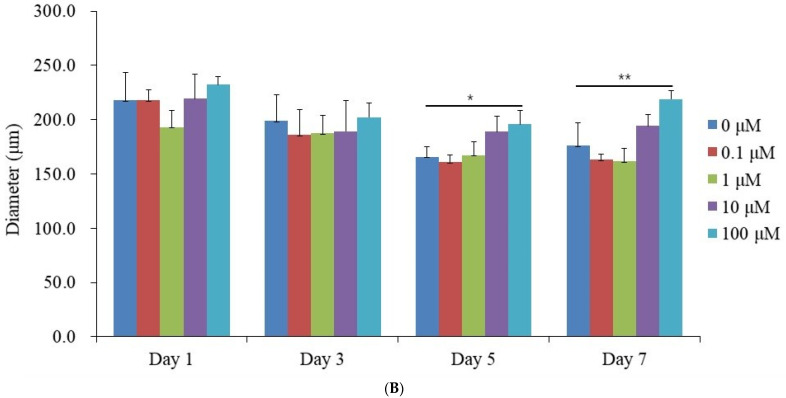
Morphological evaluation. (**A**) The morphology of the stem-cell spheroids at Days 1, 3, 5, and 7. The provided scale bar represents a length of 200 μm. (**B**) The diameter of the stem cell spheroids on Days 1, 3, 5, and 7. * *p* < 0.05 compared to the 0 μM group on Day 5. ** *p* < 0.05 compared to the 0 μM group on Day 7.

Figure 1A illustrates the morphological alterations observed in spheroids subjected to dexamethasone treatment at concentrations ranging from 0 μM to 100 μM, including intermediate concentrations of 0.1 μM, 1 μM, and 10 μM, over a period of Days 1, 3, 5, and 7. Throughout this seven-day observation period, the spheroids maintained their overall structural integrity, with no significant morphological deviations detected. Although minor variations in spheroid size were noted between Day 1 and Day 7, the characteristic rounded shape of the spheroids was consistently preserved. The diameters of the spheroids measured on Days 1, 3, 5, and 7 are summarized in Figure 1B. On Day 1, the recorded spheroid diameters for each dexamethasone concentration were as follows: 217.9 ± 25.4 µm (control, 0 μM), 218.2 ± 9.1 µm (0.1 μM), 193.3 ± 15.5 µm (1 μM), 219.4 ± 22.4 µm (10 μM), and 232.1 ± 7.5 µm (100 μM). No statistically significant differences were observed among the groups at this time point (*p* > 0.05). Similarly, spheroid diameters on Day 3 showed no significant variations across treatment conditions (*p* > 0.05). However, a statistically significant increase in spheroid size was observed in the 100 μM group on Days 5 and 7 (*p* < 0.05).

### 3.2. Assessment of Cell Viability

**Figure 2:**
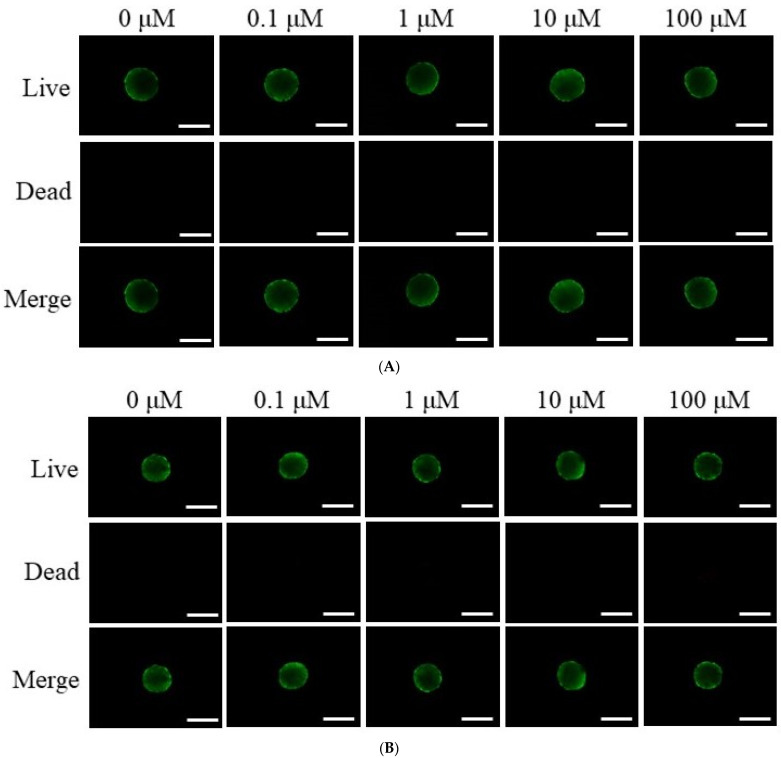

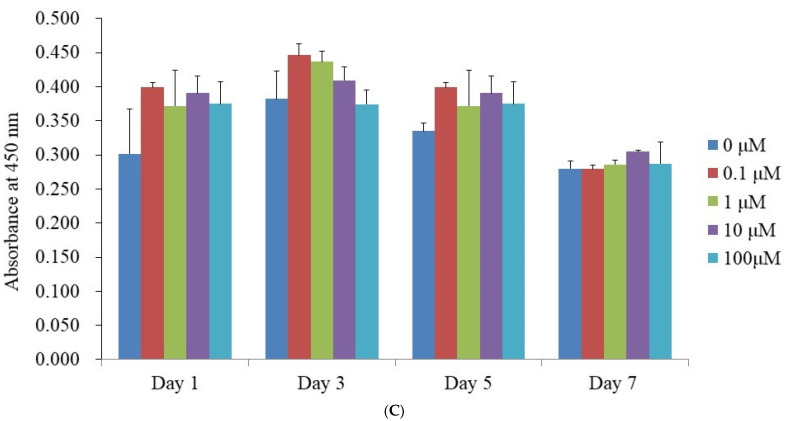
Composite images illustrating the viability of stem cell spheroids. (**A**) Representative merged images depicting live and dead cells within spheroids on Day 1, with a scale bar of 200 μm. (**B**) Composite visualization of live, dead, and merged spheroid images captured on Day 3. (**C**) Quantitative assessment of cellular viability conducted using the CCK-8 assay on Days 1, 3, 5, and 7.

Figure 2A,B present a qualitative evaluation of stem cell viability on Days 1 and 3, utilizing the Live/Dead Kit assay. As depicted in Figure 2A, the majority of stem cells displayed a rounded morphology with pronounced green fluorescence on Day 3, indicative of their viability. Similarly, Figure 2B demonstrates sustained green fluorescence following extended incubation, confirming stable cell survival through Day 3. A quantitative analysis of cell viability across multiple time points (Days 1, 3, 5, and 7) is illustrated in Figure 2C. As shown in Figure 2C, cell viability, as assessed by absorbance at 450 nm, remained consistently high across all dexamethasone concentrations. On Day 1, the 0.1 μM group demonstrated the highest absorbance (0.399 ± 0.008), followed by the 10 μM (0.391 ± 0.025), 1 μM (0.372 ± 0.052), and 100 μM (0.375 ± 0.033) groups, whereas the control exhibited the lowest value (0.302 ± 0.066). Similar trends were observed on Day 3, with the 0.1 μM group maintaining the highest viability (0.410 ± 0.040) and the control group showing the lowest (0.311 ± 0.045). No significant differences were observed among the groups (*p* > 0.05).

### 3.3. Alkaline Phosphatase Activity Levels and Calcium Deposition Extent

**Figure 3:**
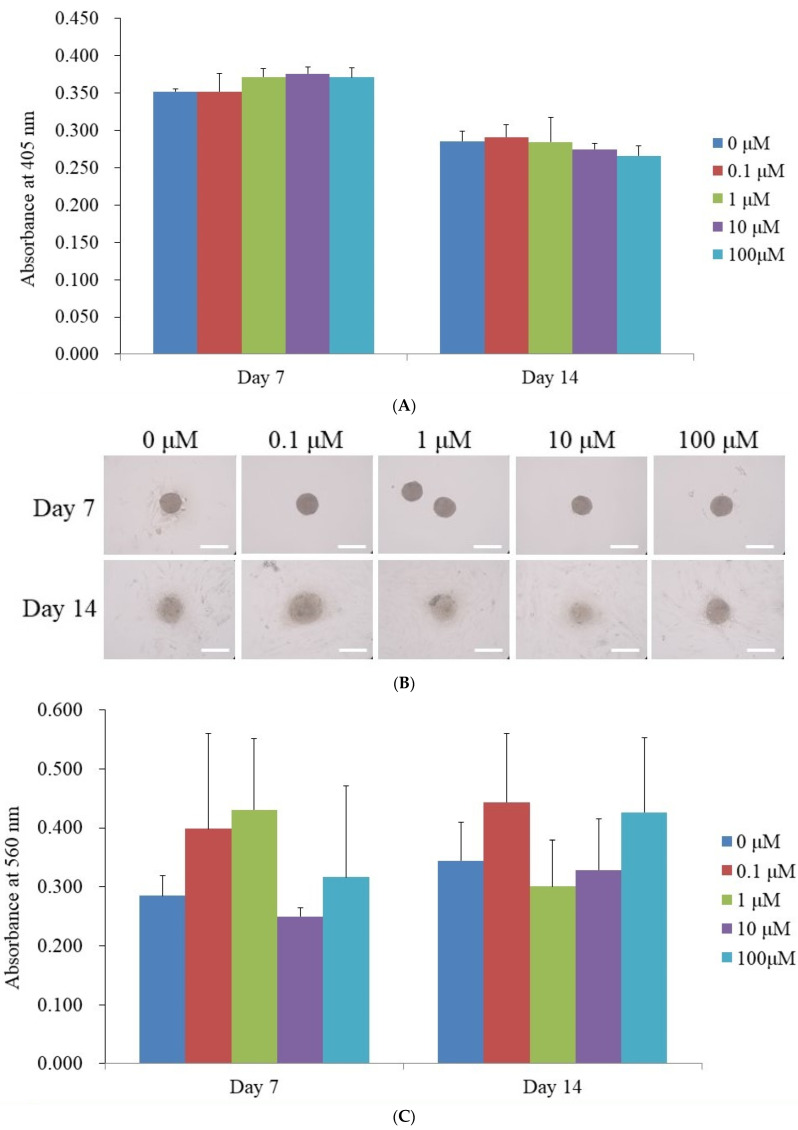
Osteogenic maturation of stem cell spheroids. (**A**) Alkaline phosphatase activity levels on Days 7 and 14. (**B**) Microscopic visualization of Alizarin Red S staining results on Days 7 and 14, with a scale bar of 200 μm. (**C**) Quantitative analysis of Alizarin Red S staining.

The findings of the ALP activity assay performed on Days 7 and 14 are summarized in Figure 3A. Absorbance measurements taken at 405 nm on Day 7 were recorded as follows: 0.352 ± 0.004 for the control (0 μM), 0.352 ± 0.024 for 0.1 μM, 0.372 ± 0.011 for 1 μM, 0.376 ± 0.009 for 10 μM, and 0.371 ± 0.013 for 100 μM. While the average absorbance values in the 1 μM, 10 μM, and 100 μM groups were slightly elevated compared to the control, these differences were not statistically significant (*p* > 0.05). Similarly, ALP activity on Day 14 did not show a significant deviation from the untreated control group.

Distinct calcium deposits were visible in all groups at both time points, as depicted in Figure 3B. The relative calcium deposition levels measured on Day 7 were 0.285 ± 0.034 (0 μM), 0.399 ± 0.161 (0.1 μM), 0.431 ± 0.121 (1 μM), 0.250 ± 0.014 (10 μM), and 0.316 ± 0.155 (100 μM), as illustrated in Figure 3C. On Day 14, the corresponding calcium deposition values were 0.345 ± 0.065 for 0 μM, 0.444 ± 0.116 for 0.1 μM, 0.301 ± 0.078 for 1 μM, 0.329 ± 0.086 for 10 μM, and 0.427 ± 0.127 for 100 μM.

### 3.4. Evaluation of RUNX2 and COL1A1 Quantitative Real-Time Polymerase Chain Reaction

**Figure 4:**
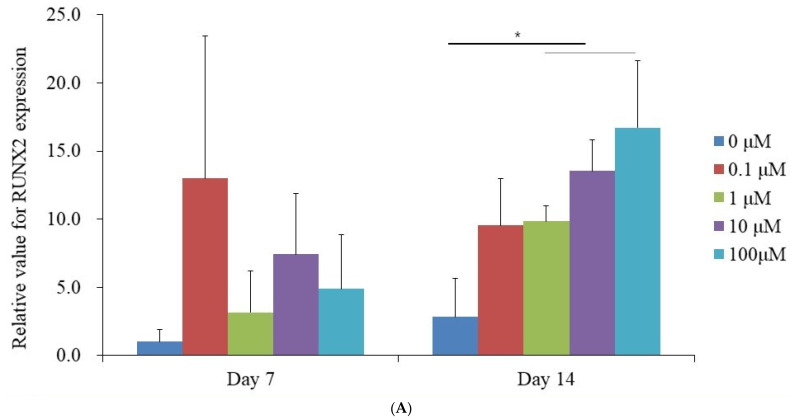

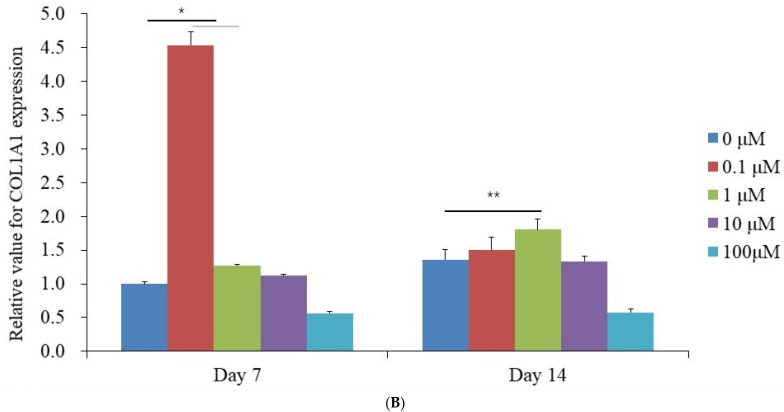
Quantification by real-time polymerase chain reaction on Days 7 and 14. (**A**) Expression of RUNX2. * *p* < 0.05 compared to the 0 μM group on Day 14. (**B**) Expression of COL1A1. * *p* < 0.05 compared to the 0 μM group on Day 7. ** *p* < 0.05 compared to the 0 μM group on Day 14.

qPCR analysis was performed to quantify the mRNA expression levels of RUNX2 and COL1A1 on Days 7 and 14. The mRNA expression of RUNX2 measured on Day 7 exhibited the following values for each dexamethasone concentration: 1.000 ± 0.915 (0 μM, control), 13.007 ± 10.423 (0.1 μM), 3.152 ± 3.054 (1 μM), 7.440 ± 4.436 (10 μM), and 4.875 ± 3.973 (100 μM). No statistically significant differences were observed among the groups (*p* > 0.05) (Figure 4A). On Day 14, an upward trend in RUNX2 mRNA levels was observed, with values recorded as 2.830 ± 2.843 (0 μM), 9.544 ± 3.408 (0.1 μM), 9.851 ± 1.138 (1 μM), 13.535 ± 2.311 (10 μM), and 16.710 ± 4.937 (100 μM), demonstrating statistical significance (*p* < 0.05). Similarly, the mRNA expression of COL1A1 on Day 7 was quantified as 1.000 ± 0.025 (0 μM, control), 4.531 ± 0.197 (0.1 μM), 1.268 ± 0.025 (1 μM), 1.122 ± 0.016 (10 μM), and 0.561 ± 0.029 (100 μM), showing a significant increase compared to the control (*p* < 0.05) (Figure 4B). By Day 14, COL1A1 expression continued to rise, with measured values of 1.355 ± 0.148 (0 μM, control), 1.497 ± 0.199 (0.1 μM), 1.804 ± 0.157 (1 μM), 1.335 ± 0.070 (10 μM), and 0.575 ± 0.048 (100 μM), confirming statistical significance (*p* < 0.05).

## 4. Discussion

This study examined the impact of dexamethasone on the structural integrity, cellular viability, and osteogenic differentiation of stem cell spheroids. These findings extend previous research that has extensively investigated dexamethasone’s role in modulating mesenchymal stem cell behavior and differentiation under various experimental conditions. The concentration of dexamethasone used in prior studies varied significantly based on the experimental design. This report demonstrated that morphological stability was maintained throughout the tested period. For instance, rat bone marrow-derived and muscle tissue-derived stem cells cultured in media with or without 10 μM dexamethasone demonstrated their efficacy in promoting osteogenesis [30]. Other studies employed dexamethasone at 1 and 100 nM for chondrogenesis [31], while higher concentrations, such as 1 μM, were shown to negatively impact cell activity by promoting apoptosis, increasing oxidative stress, and disrupting mitochondrial dynamics [32]. Additionally, studies examining concentrations between 2.55 μM and 7.64 μM over 24–48 h found that dexamethasone preserved mesenchymal stem cell morphology and viability without inducing cytotoxicity [3]. These findings collectively underscore the necessity for dose optimization to achieve therapeutic benefits without adverse effects. In this study, dexamethasone concentrations of 0.1 μM, 1 μM, 10 μM, and 100 μM did not significantly impact the viability of stem cells cultured in three-dimensional spheroid models. This observation highlights the potential of spheroid culture systems to mimic in vivo environments, possibly enhancing cellular resilience against stress. In another report, the effects of various dexamethasone concentrations, ranging from 50 nM to 10 μM, showed that dexamethasone influenced spheroid formation in a concentration-dependent manner, with higher concentrations disrupting aggregation and leading to the disassembly of cell aggregates in culture dishes [33]. Furthermore, RUNX2 expression was upregulated at 1 μM, 10 μM, and 100 μM, while COL1A1 expression was significantly upregulated at 0.1 μM and 1 μM. These concentrations appeared to improve the functional properties of the spheroids, consistent with dexamethasone’s known role in promoting osteogenesis. Calcium deposition, visualized and quantified through Alizarin Red S staining, represents late-stage osteogenic maturation, corresponding to the mineralization phase of bone tissue development. The increased calcium accumulation, although not statistically significant across groups, suggests that dexamethasone may promote matrix mineralization in 3D GMSC spheroids, which is crucial for the mechanical strength and functional integrity of regenerated bone.

The evaluation of ALP activity, a crucial indicator of osteogenic differentiation, confirmed that dexamethasone promotes early-stage bone formation [34]. ALP is prominently expressed during osteoblast differentiation and serves as a fundamental measure for assessing osteogenic potential in vitro [27]. The increased ALP activity observed in this study is consistent with previous findings, reinforcing dexamethasone’s capacity to initiate osteogenesis and its potential application in bone tissue engineering [35,36]. The analysis of mRNA expression for RUNX2 and COL1A1 provided further insights into the molecular mechanisms underlying dexamethasone-induced osteogenesis. RUNX2, a pivotal transcription factor, regulates osteoblast differentiation and skeletal growth by activating downstream genes [37]. COL1A1 encodes type I collagen, a vital structural component of the bone matrix that contributes to its mechanical strength and quality [38]. The elevated expression of COL1A1 suggests that dexamethasone may enhance the structural integrity of newly formed bone, thereby accelerating recovery and improving therapeutic outcomes. The significant upregulation of RUNX2 and COL1A1 observed in this study indicates that dexamethasone enhances the transcriptional network essential for bone formation [39]. Previous studies have demonstrated that stem cell spheroids can promote functional bone regeneration in vivo by enhancing osteogenic differentiation and vascularization [40,41], supporting the potential translational application of GMSC spheroids treated with dexamethasone for bone tissue engineering and regenerative therapies. These findings highlight the clinical significance of dexamethasone treatment, suggesting its potential to improve outcomes in bone grafting, dental implants, and fracture healing. Its dose-dependent effects present opportunities for personalized therapeutic strategies tailored to patient-specific needs, such as severe bony defects or delayed healing. Furthermore, combining dexamethasone with osteoinductive agents like bone morphogenetic proteins or vascular endothelial growth factors could enhance its therapeutic efficacy by simultaneously promoting osteogenesis and angiogenesis.

Despite these promising results, several limitations inherent in this study must be acknowledged. Three-dimensional stem cell spheroids exhibit considerable potential for diverse tissue engineering applications; however, current spheroid fabrication methods face challenges such as diminished cell viability due to limited oxygen availability at the core and nonspecific differentiation resulting from the complex post-transplantation environment [42]. Additionally, the administration of dexamethasone may impede spheroid formation, necessitating careful regulation of its application timing [43].

As this investigation was conducted entirely in vitro, the findings require in vivo validation to ascertain the long-term effects of dexamethasone on bone regeneration and remodeling. Furthermore, while this study concentrated on RUNX2 and COL1A1 as primary markers, future research should encompass a broader spectrum of osteogenic markers, including osteocalcin, osteopontin, and bone sialoprotein, to achieve a more comprehensive understanding of dexamethasone’s impact on osteogenesis [44].

Bone formation is intricately modulated by the interaction between osteogenesis and angiogenesis, and the potential for multidirectional cell differentiation in stem cell-based spheroids may offer promising future applications [45]. Future investigations should explore the synergistic potential of dexamethasone with advanced tissue engineering technologies, such as three-dimensional bioprinting and microfluidic systems, to optimize its delivery and efficacy. These approaches, combined with finely tuned dosage protocols, could facilitate the advancement of personalized regenerative medicine, providing effective solutions for bone repair and regeneration.

## 5. Conclusions

This study investigated the impact of dexamethasone on stem cell spheroids, focusing on their ability to preserve structural integrity, enhance cell viability, and promote osteogenic differentiation. The findings indicated that dexamethasone facilitated cellular survival and significantly increased the expression of RUNX2 and COL1A1 in stem cell spheroids. The incorporation of stem cells with dexamethasone presents a promising strategy for regenerative medicine and therapeutic applications. Future research should concentrate on optimizing dosage levels, developing efficient delivery systems, and assessing the long-term outcomes of these treatments.

## Figures and Tables

**Table 1:** The primer sequences used for real-time quantitative polymerase chain reaction (qPCR).

Gene	Forward Primer (5′ → 3′)	Reverse Primer (5′ → 3′)
RUNX2	AAGTGCGGTGCAAACTTTCT	TCTCGGTGGCTGCTAGTGA
COL1A1	CCAGAAGAACTGGTACATCAGCAA	TGGTTTCTTCTCCTCTGCGC
β-actin	TGGCACCCAG CACAATGAA	CTAAGTCATAGTCCGCCTAGAAGCA

## Data Availability

The original contributions presented in this study are included in the article; further inquiries can be directed to the corresponding author.
